# Clinical Study on Systemic Lupus Erythematosus Complicated with Knee Bone Infarction

**DOI:** 10.1155/2022/7025811

**Published:** 2022-07-18

**Authors:** Gui-Qi Zhu, Hong-Xia Qiu, Xin-mei Ma, Mei-Xia Liu

**Affiliations:** ^1^Department of Rheumatology, Zaozhuang Municipal Hospital, Zaozhuang 277000, China; ^2^Department of Rheumatology, Xi'an Fifth Hospital, Xi'an 710000, China; ^3^Department of Physiotherapy, Zaozhuang Municipal Hospital, Zaozhuang 277000, China

## Abstract

**Objective:**

The present study aims to (1) analyze the clinical characteristics and related influencing factors of knee bone infarction in systemic lupus erythematosus (SLE) and (2) improve the understanding of SLE complicated with knee bone infarction.

**Methods:**

The data of patients with SLE complicated with knee bone infarction were retrospectively analysed; patients with SLE during the same period who matched in age, gender, and disease duration were selected as control subjects, with a 1 : 1 ratio with the SLE group. The clinical data were collected to analyze the risk factors for SLE complicated with knee bone infarction.

**Results:**

In a total of 36 (6.4%) of 563 patients aged 19–33 (25.8 ± 4.8) years who had SLE during the same period, the disease was complicated with knee bone infarction. The diagnosis of knee bone infarction was made at an SLE duration of 7–65 (26.2 ± 15.7) months. During the SLE course, knee bone infarction occurred within 1 year in 6 cases (16.7%), within 1–5 years in 28 cases (77.8%), and in >5 years in 2 cases (5.6%). Raynaud's phenomenon incidence and anti-nRNP antibody positivity were significantly higher in the knee bone infarction group than in the control group (*P* < 0.01 and *P* < 0.05, respectively). The cumulative glucocorticoid dose at 1, 3, and 6 months was significantly higher in the knee bone infarction group than in the control group (*P* < 0.05). SLE complicated with knee necrosis had a statistically significant rank correlation with Raynaud's phenomenon (*r* = 0.445, *P* < 0.001), anti-nRNP antibody (*r* = 0.309, *P*=0.008), and renal injury (*r* = 0.252, *P*=0.032). The multivariate analysis of SLE complicated with knee bone infarction showed that Raynaud's phenomenon was an independent influencing factor for the complicated knee bone infarction in SLE patients (OR = 4.938, *P*=0.004), and the probability of SLE complicated with knee bone infarction in Raynaud's phenomenon positive patients was 4.938 times that of Raynaud's phenomenon negative patients.

**Conclusions:**

The risk of knee bone infarction was relatively high in patients with SLE within a 5-year disease course and in young patients. The risk factors were Raynaud's phenomenon, anti-nRNP antibody positivity, and early high-dose glucocorticoid therapy.

## 1. Introduction

Systemic lupus erythematosus (SLE) is a chronic autoimmune disease with complicated clinical manifestations that may involve multiple systems and organs [1, 2] and often manifests with functional and morphological microvascular impairment [3]. A serious complication of SLE is bone infarction. At present, studies at home and abroad are primarily limited in SLE complicated with femoral head necrosis, while there are few reports regarding knee bone infarction. The incidence and risk factors are unclear for SLE complicated with knee bone infarction, which seriously affects the daily life of patients [4]. To investigate the risk factors affecting SLE incidence, the clinical and laboratory characteristics of SLE complicated with knee bone infarction were analysed in the present study, which might provide a specific basis for clinical practice and increase the understanding of SLE complicated with knee bone infarction.

## 2. Materials and Methods

### 2.1. General Data

A total of 563 patients with SLE hospitalized in the Department of Rheumatology, Zaozhuang Municipal Hospital, from June 2011 to June 2021 were selected as the study subjects. All cases met the SLE classification diagnostic criteria revised by the American College of Rheumatology in 1997 [5]. There were 36 cases of SLE complicated with knee bone infarction, accounting for 6.4% of the total hospitalized patients; all cases were confirmed via magnetic resonance imaging (MRI), and the patients had no definite history of trauma.

Based on the annual number of cases with knee bone infarction from 2012 to 2020, 36 patients matching in age, gender, and disease course were randomly selected from hospitalized patients with SLE in the corresponding years. The ratio of the knee bone infarction group and the control group was 1 : 1.

All enrolled patients met the following criteria: (1) patients with no history of trauma or alcoholism; (2) patients with complete auxiliary examination data; and (3) patients with a definite glucocorticoid dosage.

This study was conducted with approval from the Ethics Committee of Zaozhuang Municipal Hospital. Consent was received from the study participants. This study was conducted in accordance with the Declaration of Helsinki.

### 2.2. Study Methods

The patients were divided into the knee bone infarction group and the control group according to whether they had knee bone infarction. The general characteristics, clinical manifestations, laboratory results, and SLE disease activity index (SLEDAI) scores were compared between the two groups (the data were collected at the time of bone infarction clinical diagnosis in the bone infarction group and at the corresponding time point in the control group).

The general characteristics included patient gender, age, and disease course; the clinical manifestations mainly included oral ulcer, Raynaud's phenomenon, skin vasculitis, facial erythema, photosensitivity, hypertension, and renal injury; the related examinations included the levels of 17 items of the antinuclear antibody (ANA) spectrum (including anti-nRNP antibody, anti-SSA antibody, anti-SSB antibody, antinucleosome antibody, anti-dsDNA antibody, antiribosomal P antibody, and anti-Sm antibody, which were detected by the immunofluorescence method), anticardiolipin antibody (aCL), complement 3 (C3), C4, D-dimer, and lipoprotein (the serum levels of low-density lipoprotein cholesterol (LDL-C), total cholesterol (TC), and triglycerides (TG) under a 12 h fasting condition); and the treatment features mainly included the initial dose, cumulative dose, and conduction of glucocorticoid pulse therapy.

### 2.3. Statistical Analysis

All the data collected in this study were analysed using the SPSS 20.0 software. The measurement data were expressed as mean ± standard deviation (SD), and the comparisons were examined by Student's *t*-test and the Mann–Whitney test. The categorical data were expressed as *n* (%), and the differences between the two groups were examined by the *χ*^2^ test or Fisher's exact test. *P* < 0.05 was considered statistically significant.

## 3. Results

### 3.1. General Characteristics

The knee bone infarction group comprised 2 males and 34 females aged 19–33 (25.8 ± 4.8) years. The SLE course was 7–65 (26.2 ± 15.7) months at the time of knee bone infarction diagnosis. During the SLE course, knee bone infarction occurred within 1 year in 6 cases (16.7%), within 1–5 years in 28 cases (77.8%), and in >5 years in 2 cases (5.6%). The control group comprised 3 males and 33 females aged 18–36 (26.4 ± 6.9) years. The SLE course was 8–64 (27.8 ± 14.9) months. There were no significant differences in age, gender, and disease course between the two groups (*P* > 0.05). Femoral head necrosis occurred in a total of 6 patients within 0–5 years after the occurrence of knee bone infarction; none of the patients had femoral head necrosis before the occurrence of knee bone infarction.

### 3.2. Characteristics of Knee Bone Infarction in Patients with Systemic Lupus Erythematosus

Of the 36 patients with knee bone infarction, 12 (33.4%) had bilateral knee bone infarction (manifesting as only unilateral knee pain in 4 patients) and 24 (66.6%) had unilateral knee bone infarction. A total of 31 cases (86.1%) had the lower femur infarction and 22 cases (61.1%) had infarction of the upper tibia. All patients had effusion of the affected knee joint. Paroxysmal pain was the main manifestation in the early stage of the disease. The pain occurred during walking and weight-bearing, and it would be relieved after rest. In the late stage of the disease, persistent pain that was not accompanied by pain in the rest of the joint occurred.

### 3.3. Comparison of Clinical Features between the Two Groups

The incidence of Raynaud's phenomenon and renal injury was significantly higher in the knee bone infarction group than in the control group (*P*=0.002 and *P*=0.03, respectively). The incidence of arthritis was also significantly higher in the knee bone infarction group than in the control group; however, the difference was not statistically significant (*P*=0.08). There was no significant difference in the incidence of their clinical manifestations, such as recurrent oral ulcer, cutaneous vasculitis, photosensitivity, hyperglycemia, hypertension, malar rash, neuropsychiatric, leukopenia, autoimmune hemolytic anemia (AIHA), and thrombocytopenia between the two groups (*P* > 0.05; Table 1). The SLEDAI score was 5.22 ± 1.79 at the time of knee bone infarction diagnosis in the knee bone infarction group and 4.97 ± 1.58 at the corresponding time in the control group; there was no statistically significant difference between the two groups (*t* = 1.63; *P*=0.53).

### 3.4. Comparison of Laboratory Indicators between the Two Groups

The anti-nRNP antibody positivity was significantly higher in the knee bone infarction group than in the control group (72.2% *vs.* 41.7%, *P*=0.007). The anti-SSA antibody positivity was also higher in the knee bone infarction group than in the control group; however, the differences were not statistically significant (63.9% *vs.* 41.7%, *P* > 0.05). There were no significant differences between the knee bone infarction group and the control group, in the respective positivity of the anti-SSB antibody (25.0% *vs.* 27.8%), anti-nucleosome antibody (30.6% *vs* 36.1%), anti-dsDNA antibody (44.5% *vs.* 38.9%), anti-ribosomal *P* antibody (33.3% *vs.* 36.1%), anti-Sm antibody (27.8% *vs.* 25.0%), anti-B2 glycoprotein antibody (30.6% *vs.* 16.7%), lupus anticoagulant (36.1% *vs.* 25.0%), and aCL antibody (25.0% *vs.* 16.7%) or in the levels of LDL-C, TG, TC, plasma D-dimer, C3, and C4 between the two groups (*P* > 0.05; Table 2).

### 3.5. Correlation with Glucocorticoids

The patients in the two groups who had a definite diagnosis of SLE were immediately treated with hormone therapy; there was no significant difference in glucocorticoid pulse therapy between the two groups (*P* > 0.05; Table 2). The cumulative glucocorticoid doses at 1, 3, and 6 months were significantly higher in the knee bone infarction group than in the control group (*P*=0.04, *P*=0.03, and *P*=0.002, respectively). There was no significant difference in glucocorticoid pulse therapy and the total cumulative dose (the cumulative glucocorticoid dose at the time of bone infarction occurrence in the knee bone infarction group and at the corresponding time point in the control group) between the two groups (*P* > 0.05; Table 3).

### 3.6. Comparison of Bone Infarction in Patients with Juvenile-Onset Systemic Lupus Erythematosus (jSLE) and Adult-Onset SLE(aSLE)

Bone infarction was more prominent in the aSLE group (*n* = 34, 6.8% in the aSLE group vs. *n* = 2, 3.4% in the jSLE group), but the difference between them was not statistically significant (*P*=0.46; Table 4).

### 3.7. Correlation Analysis for SLE Complicated with Knee Bone Infarction

SLE complicated with knee necrosis had a statistically significant rank correlation with Raynaud's phenomenon (*r* = 0.445, *P* < 0.001), anti-nRNP antibody (*r* = 0.309, *P*=0.008), and renal injury (*r* = 0.252, *P*=0.032). The positive likelihood ratios of the three factors ranged from 1.65 to 2.68, and the negative likelihood ratios ranged from 0.39 to 0.54, as shown in Table 5.

### 3.8. Multivariate Analysis of SLE Complicated with Knee Bone Infarction

Whether SLE patients were complicated with knee bone infarction was taken as the dependent variable, and influencing factors whose *P* value in the univariate analysis was less than 0.05 were taken as independent variables in the binary logistic regression model. The results showed that Raynaud's phenomenon was an independent influencing factor for the complicated knee bone infarction in SLE patients (*P*=0.004), and the probability of SLE complicated with knee bone infarction in Raynaud's phenomenon positive patients was 4.938 times that of Raynaud's phenomenon negative patients, as shown in Table 6.

## 4. Discussion

Osteonecrosis (ON) results from bone circulation destruction, osteoblast, and local bone marrow tissue death caused by various factors. Generally, ON refers to the ON of the epiphyseal or subarticular surface. Bone infarction, also known as bone marrow infarction, refers to bone ischemic necrosis in the backbone and metaphysis (usually occurring in the lower end of femur and the upper end of tibias and rarely involving the articular surface) [6]. In this study, a total of 31 cases (86.1%) had the lower femur infarction and 22 cases (61.1%) had infarction of the upper tibia, and both bone infarctions did not involve the articular surface.

In addition to nondestructive arthritis, SLE can also lead to bone infarction, which is a serious complication of the disease [7–10]. In the bone marrow lumen of the long tubular bones located in the extremities, the thin and small nutrient vessels with few branches can easily lead to bone marrow ischemia necrosis and bone infarction. Common symptoms of knee bone infarction are metaphysis, knee joint effusion, mobility disorder [11], and dull or swelling pain in the backbone. MRI is a sensitive and effective examination method for the early detection of bone infarction. Map plate-like lesions are typical MRI manifestations of bone infarction, while bilateral signs are considered to be relatively specific MRI manifestations of bone infarction [12]. The results of the present study showed that the incidence of SLE complicated with knee bone infarction was approximately 6.4%. Among the 36 patients with knee bone infarction, 34 (94.4%) developed knee bone infarction within 5 years of the disease course, and all patients had effusion of the affected knee.

It has been reported in literature [12] that when infarction occurred, the amount of effusion increased due to local venous return obstruction. Articular effusion might also be the cause of clinical pain. All cases in the present group were complicated with a small or moderate amount of articular effusion; this is consistent with the relevant literature. Most of the patients in the knee bone infarction group were aged 19–33 (25.8 ± 4.8, primarily <30) years and had a disease duration of 8–64 (27.8 ± 14.9) months from the diagnosis of SLE to the occurrence of knee bone infarction. Thus, knee bone infarction might mostly occur in young patients with SLE and usually occurs within the first 5 years after onset.

Glucocorticoids are the basic drugs for SLE treatment, and a large number of studies believe that glucocorticoid therapy is the main factor leading to ON in patients with SLE. Glucocorticoids can prolong the life cycle of osteoclasts by inducing apoptosis of osteoblasts and osteocytes. They can also increase the bone marrow fat content as well as intramedullary pressure, resulting in reduced blood flow, ischemia and hypoxia of bone cells, and osteonecrosis [13, 14]. Studies have found that patients receiving glucocorticoid treatment have a significantly increased risk of ON when the dosage of prednisone is >40 mg/day [15, 16]. The present study revealed that the cumulative glucocorticoid dose at 3 and 6 months was significantly higher in the knee bone infarction group than in the control group; there was no statistically significant difference in the total cumulative dose. All 28 patients had bone infarction of the knee joint during the first 5 years of SLE; this suggests the possibility of a correlation with the extensive administration of glucocorticoids during the induction remission period, which might be a risk factor for SLE complicated with bone infarction. Massardo et al. [17] studied 190 patients with SLE and found that methylprednisolone pulse therapy was a risk factor for ON; this suggested that ON was a long-term adverse reaction of methylprednisolone pulse therapy. However, no significant difference was found between the two groups in the present study; this may be due to the low number of observed cases.

Most researchers believe that the occurrence of ON is affected by multiple factors and cannot be explained by a single risk factor. Apart from glucocorticoids, SLE itself is also correlated with ON occurrence [18–20]. Through further clinical analysis of the 36 patients with SLE complicated with knee bone infarction included in the present study, it was found that glucocorticoids were not the only factor leading to knee bone infarction, but also that Raynaud's phenomenon, positive anti-nRNP antibody, and renal injury were correlated with knee bone infarction. However, there was no correlation with anti-dsDNA antibody, anti-Sm antibody, and the SLEDAI score at the clinical diagnosis of bone infarction to the knee bone infarction.

Vasculitis is the basic lesion of SLE; here, a large amount of immune complex is deposited on the walls of small blood vessels, causing signs of small vessel degeneration, such as vasculitis, Raynaud's phenomenon, and oral ulcer under the synergistic effect of the complement. This pathological change appears in extremities, but also in other blood vessels such as viscera, thickening of the vascular intima, affected tissue blood supply, etc. The present study found that the incidence of Raynaud's phenomenon was significantly higher in the knee bone infarction group than in the control group. In most cases, other symptoms were relieved by the time of bone infarction; however, Raynaud's phenomenon persisted. This further indicated that Raynaud's phenomenon might be a risk factor for knee bone infarction and also suggested a possible correlation between knee bone infarction and the underlying vascular lesions.

Anti-SSA antibody is not a specific antibody and is independently associated with SLE when compared with Sjögren's syndrome and other systemic autoimmune diseases. In a study of atherosclerosis associated with Sjögren's syndrome, Vaudo et al. [21] found that the serum anti-SSA antibody positivity was higher in patients with carotid intima-media thickness than in patients without carotid intima-media thickness; they further found that the antibody was an independent predictor for carotid intima-media thickness. In their research, Yao et al. found that patients with positive anti-SSA antibody were prone to the occurrence of Raynaud's phenomenon, pulmonary hypertension, limb necrosis, nervous system involvement, oral ulcers, and other clinical symptoms [22]. It has been reported that the SSA antigen intracranial vascular expression selectively increases in patients with Sjögren's syndrome and SLE, suggesting that the SSA antigen plays a certain role in the pathogenesis of vascular injury [23]. The injury of nutrient vessels in the bone marrow cavity induces the occurrence of bone infarction. The positive rate of anti-SSA antibodies in the knee bone infarction group was 63.9%, which was significantly higher than 41.7% in the control group. Although there was no statistical significance between the two groups, whether anti-SSA antibody is positively related to bone infarction requires more data to confirm [24].

Vascular necrosis was more frequently reported in patients with adult-onset lupus compared to childhood-onset lupus according to Artim-Esen et al. (30.9% in the aSLE group vs. 16% in the jSLE group; *P*=0.003). However, there are limited case series reporting the prevalence of knee bone infarction in SLE. Through the retrospective analysis of 563 SLE patients, we found that the incidence of bone infarction of the knee in the jSLE group was 6.8%, while that in the aSLE group was 3.4%. Bone infarction of the knee was more prominent in the aSLE group, but the difference between them was not statistically significant (*P* > 0.05).

Since the early clinical symptoms of knee bone infarction are not obvious, the condition is prone to missed diagnosis. A total of 2 patients in this study presented with unilateral knee pain, and an MRI examination revealed extensive bilateral knee bone infarction. Therefore, during the early stage of SLE when the patients are being administered with a large dose of glucocorticoids, clinicians should pay attention to the occurrence of knee pain and other clinical symptoms, improve the diagnosis awareness of knee bone infarction, and comprehensively better the MRI examination, with the aim to detect, diagnose, and treat bone infarction at an early stage.

In conclusion, the incidence of knee bone infarction was relatively high. The infarction usually occurred in young patients with SLE. Furthermore, the risk of knee bone infarction might be relatively high within 5 years of the disease course. Most patients with SLE combined with knee bone infarction had Raynaud's phenomenon and were positive for the anti-nRNP antibody; most of these patients had been treated with a high glucocorticoid dose.

## Figures and Tables

**Table 1:** Comparison of the related indicators between the two groups.

	Bone infarction group	Control group	*χ* ^2^	*P*
*n*	36	36		
Recurrent oral ulcer	14 (38.9%)	9 (25%)	1.60	0.21
Raynaud's phenomenon	27 (75.0%)	11 (30.6%)	14.27	<0.001
Cutaneous vasculitis	7 (19.5%)	5 (13.9%)	0.40	0.53
Photosensitivity	8 (22.2%)	10 (27.8%)	0.30	0.59
Hypertension	4 (11.1%)	3 (8.3%)	0.16	0.69
Diabetes mellitus	2 (5.6%)	4 (11.1%)	0.70	0.39
Renal injury	20 (55.6%)	11 (30.6%)	4.59	0.03
Malar rash	13 (36.1%)	11 (30.6%)	0.25	0.62
Arthritis	16 (44.4%)	9 (25%)	3.00	0.08
Neuropsychiatric	6 (16.7%)	4 (11.1%)	0.47	0.50
Leukopenia	17 (47.2%)	21 (58.3%)	0.89	0.35
AIHA	8 (47.2%)	7 (19.5%)	0.08	0.77
Thrombocytopenia	17 (47.2%)	19 (52.8%)	0.22	0.64

**Table 2:** Comparison of the laboratory indicators between the two groups.

	Bone infarction group	Control group	*χ* ^2^/*t*	*P*
*n*	36	36		
Anti-SSA antibody	23 (63.9%)	15 (41.7%)	3.57	0.06
Anti-SSB antibody	9 (25.0%)	10 (27.8%)	0.07	0.79
Anti-nRNP antibody	26 (72.2%)	15 (41.7%)	6.85	0.01
aCL	9 (25.0%)	6 (16.7%)	0.76	0.38
Anti-B2 glycoprotein antibody	11 (30.6%)	6 (16.7%)	1.93	0.17
Lupus anticoagulant	13 (36.1%)	9 (25.0%)	1.05	0.31
Antinucleosome antibody	11 (30.6%)	13 (36.1%)	0.25	0.62
Anti-dsDNA antibody	16 (44.5%)	14 (38.9%)	0.23	0.63
Ribosomal *P* antibody	12 (33.3%)	13 (36.1%)	0.06	0.80
Anti-Sm antibody	10 (27.8%)	9 (25.0%)	0.07	0.79
TG (mmol/L)	1.56 ± 0.34	1.51 ± 0.33	0.66	0.51
TC (mmol/L)	4.47 ± 0.32	4.35 ± 0.20	1.83	0.07
LDL-C (mmol/L)	2.16 ± 0.20	2.23 ± 0.27	1.34	0.19
D-dimer (ng/mL)	441.64 ± 44.90	421.14 ± 54.75	1.74	0.08
C3 (g/L)	0.88 ± 0.09	0.89 ± 0.09	0.72	0.47
C4 (g/L)	0.10 ± 0.03	0.11 ± 0.03	1.16	0.24

**Table 3:** Comparison of the dose of prednisone between patients with SLE complicated with knee bone infarction and the control group.

	Bone infarction group	Control group	*t*/*χ*^2^	*P*
*n*	36	36		
The cumulative dose of one month (mg)	1953.33 ± 498.45	1710.86 ± 486.05	2.09	0.04
The cumulative dose of 3 months (mg)	3899.83 ± 505.89	3616.28 ± 549.10	2.28	0.03
The cumulative dose of 6 months (mg)	7224.19 ± 529.42	6829.92 ± 493.69	3.27	0.002
Total cumulative dose (mg)	10142.36 ± 1090.65	9783.06 ± 1168.08	1.34	0.18
The pulse therapy (*n*)	3	2	0.22	0.64

**Table 4:** Comparison of bone infarction in patients with jSLE and aSLE.

	jSLE, *n* = 60	aSLE, *n* = 503	*χ* ^2^	*P*
Bone infarction	2 (3.4%)	34 (6.8%)	0.56	0.46

jSLE, juvenile-onset systemic lupus erythematosus; aSLE, adult-onset SLE.

**Table 5:** Correlation and likelihood ratio of three factors with SLE complicated with knee bone infarction.

	Correlation analysis		Likelihood ratio	
	*R*	*P*	Positive	Negative
Raynaud's phenomenon	0.445	<0.001	1.90	0.54
Anti-nRNP antibody	0.309	0.008	2.68	0.39
Renal injury	0.252	0.032	1.65	0.58

**Table 6:** Multivariate analysis of SLE complicated with knee bone infarction.

	*P*	OR (95% CI)
Raynaud's phenomenon	0.004	4.938 (1.659–14.698)
Renal injury	0.980	2.536 (0.842, 7.635)
Anti-nRNP antibody	0.070	2.791 (0.918, 8.485)

## Data Availability

The datasets used and/or analysed during the current study are available from the corresponding author on reasonable request.

## Abbreviations

SLE: Systemic lupus erythematosus
aCL: Anticardiolipin antibody
SLEDAI: Systemic lupus erythematosus disease activity index
ON: Osteonecrosis
LDL-C: Low-density lipoprotein cholesterol
TG: Triglyceride
TC: Total cholesterol
AIHA: Autoimmune hemolytic anemia
jSLE: Juvenile-onset systemic lupus erythematosus
aSLE: Adult-onset SLE.

## References

[1] Tsokos G. C. (2011). Systemic lupus erythematosus. *New England Journal of Medicine*.

[2] Tamirou F., Arnaud L., Talarico R. (2018). Systemic lupus erythematosus: state of the art on clinical practice guidelines. *RMD Open*.

[3] Ruaro B., Sulli A., Casabella A. (2021). Peripheral blood perfusion in patients with systemic lupus erythematosus and in primary raynaud’s phenomenon. *European journal of rheumatology*.

[4] Jeong H. J., Kim D., Cho S. K., Kim Y., Bae S. C., Sung Y. K. (2018). Clinical characteristics of multifocal osteonecrosis in Korean patients with rheumatic disease. *International journal of rheumatic diseases*.

[5] Hochberg M. C. (1997). Updating the American college of rheumatology revised criteria for the classification of systemic lupus erythematosus. *Arthritis & Rheumatism*.

[6] Lafforgue P., Trijau S. (2016). Bone infarcts: unsuspected gray areas?. *Joint Bone Spine*.

[7] Thomas J. (2020). Osteonecrosis and multifocal bone infarction in systemic lupus erythematosus. *Journal of Clinical Rheumatology*.

[8] Jin W., Yang X., Lu M. (2021 Jan 15). Juvenile-onset multifocal osteonecrosis in systemic lupus erythematosus: a case report. *Medicine*.

[9] Salesi M., Karimifar M., Mottaghi P., Sayedbonakdar Z., Karimzadeh H. (2010). A case of SLE with bilateral osteonecrosis of femoral heads and bone infarct in distal of femur. *Rheumatology International*.

[10] Ruaro B., Casabella A., Paolino S. (2020). Trabecular bone score and bone quality in systemic lupus erythematosus patients. *Frontiers of Medicine*.

[11] Boontanapibul K., Steere J. T., Amanatullah D. F., Huddleston J. I., Maloney W. J., Goodman S. B. (2020). Initial presentation and progression of secondary osteonecrosis of the knee. *The Journal of Arthroplasty*.

[12] Long B., Dong J., Song S. (2020). Imaging findings of bone infarction of the knee with X-ray and MRI. *Chinese Journal of CT and MRI*.

[13] García-Carrasco M., Mendoza-Pinto C., Escárcega R. O. (2009). Osteoporosis in patients with systemic lupus erythematosus. *Israel Medical Association Journal: IMAJ.*.

[14] Prasad R., Ibanez D., Gladman D., Urowitz M. (2007). The role of non-corticosteroid related factors in osteonecrosis (ON) in systemic lupus erythematosus: a nested case-control study of inception patients. *Lupus*.

[15] Shigemura T., Nakamura J., Kishida S. (2011). Incidence of osteonecrosis associated with corticosteroid therapy among different underlying diseases: prospective MRI study. *Rheumatology*.

[16] Kallas R., Li J., Petri M. (2020). Predictors of osteonecrosis in systemic lupus erythematosus: a prospective cohort study. *Arthritis Care & Research*.

[17] Massardo L., Jacobelli S., Leissner M., González M., Villarroel L., Rivero S. (1992). High-dose intravenous methylprednisolone therapy associated with osteonecrosis in patients with systemic lupus erythematosus. *Lupus*.

[18] Hussein S., Suitner M., Béland-Bonenfant S. (2018). Monitoring of osteonecrosis in systemic lupus erythematosus: a systematic review and metaanalysis. *Journal of Rheumatology*.

[19] Gontero R. P., Bedoya M. E., Benavente E., Roverano S. G., Paira S. O. (2015). Osteonecrosis in systemic lupus erythematosus. *Reumatología Clínica*.

[20] Faezi S. T., Hoseinian A. S., Paragomi P. (2015). Non-corticosteroid risk factors of symptomatic avascular necrosis of bone in systemic lupus erythematosus: a retrospective case-control study. *Modern Rheumatology*.

[21] Vaudo G., Bocci E. B., Shoenfeld Y. (2005). Precocious intima-media thickening in patients with primary sjögren’s syndrome. *Arthritis & Rheumatism*.

[22] Yao M., Tao S., Jun L. (2010). Clinical features of patients with systemic lupus erythematosus with anti-SSA antibody positive. *Chinese Journal of Misdiagnostics*.

[23] Shusta E. V., Yi Li J., Boado R. J., Pardridge W. M. (2003). The Ro52/SS-A autoantigen has elevated expression at the brain microvasculature. *NeuroReport*.

[24] Artim-Esen B., Şahin S., Çene E. (2017). Comparison of disease characteristics, organ damage, and survival in patients with juvenile-onset and adult-onset systemic lupus erythematosus in a combined cohort from 2 tertiary centers in Turkey. *Journal of Rheumatology*.

